# Platelet-derived growth factor receptor-alpha positive cardiac progenitor cells derived from multipotent germline stem cells are capable of cardiomyogenesis *in vitro* and *in vivo*

**DOI:** 10.18632/oncotarget.16772

**Published:** 2017-03-31

**Authors:** Bang-Jin Kim, Yong-Hee Kim, Yong-An Lee, Sang-Eun Jung, Yeong Ho Hong, Eun-Ju Lee, Byung-Gak Kim, Seongsoo Hwang, Jeong Tae Do, Myung-Geol Pang, Buom-Yong Ryu

**Affiliations:** ^1^ Department of Animal Science & Technology, Chung-Ang University, Anseong, Republic of Korea; ^2^ Laboratory of Bioimaging Probe Development, Singapore Bioimaging Consortium, Agency for Science, Technology and Research, Singapore; ^3^ Department of Internal medicine, Seoul National University, Seoul, Republic of Korea; ^4^ Bio Environment Technology Research Institute, Chung-Ang University, Anseong, Republic of Korea; ^5^ Animal Biotechnology Division, National Institute of Animal Science, Jeollabuk-do, Republic of Korea; ^6^ Department of Stem Cell and Regenerative Biology, College of Animal Bioscience and Technology, Konkuk University, Seoul, Republic of Korea; ^7^ Department of Cancer Biology, University of Pennsylvania, Philadelphia, Pennsylvania, United States of America

**Keywords:** germ-line stem cells, multipotent, testis, cardiac progenitor, differentiation

## Abstract

Cardiac cell therapy has the potential to revolutionize treatment of heart diseases, but its success hinders on the development of a stem cell therapy capable of efficiently producing functionally differentiated cardiomyocytes. A key to unlocking the therapeutic application of stem cells lies in understanding the molecular mechanisms that govern the differentiation process. Here we report that a population of platelet-derived growth factor receptor alpha (PDGFRA) cells derived from mouse multipotent germline stem cells (mGSCs) were capable of undergoing cardiomyogenesis *in vitro*. Cells derived *in vitro* from PDGFRA positive mGSCs express significantly higher levels of cardiac marker proteins compared to PDGFRA negative mGSCs. Using *Pdgfra* shRNAs to investigate the dependence of *Pdgfra* on cardiomyocyte differentiation, we observed that *Pdgfra* silencing inhibited cardiac differentiation. In a rat myocardial infarction (MI) model, transplantation of a PDGFRAenriched cell population into the rat heart readily underwent functional differentiation into cardiomyocytes and reduced areas of fibrosis associated with MI injury. Together, these results suggest that mGSCs may provide a unique source of cardiac stem/progenitor cells for future regenerative therapy of damaged heart tissue.

## INTRODUCTION

Current therapeutic approaches for end-stage heart failure are limited to pharmacological therapies, mechanical ventricular assist devices (VAD), and cardiac transplantation. Unfortunately, even the availability of a donor hearts is limited by complications associated with a lifetime of immunosuppression. Stem cell-based therapies offer a novel approach to overcome these limitations by replacing damaged or lost myocardial tissues and restore cardiac functions. Several candidate cell types used in preclinical animal models and humans studies include, embryonic stem cells (ESCs), induced pluripotent stem cells (iPSCs), neonatal cardiomyocytes, skeletal myoblasts (SKMs), endothelial progenitor cells (EPCs), and mesenchymal stem cells (MSCs) [1–4]. A consensus, however, on the ideal cell type for the treatment of heart disease has yet to be reached.

The success of stem cell therapy in cardiac regeneration relies, in part, upon identifying cell surface makers that enable reliable enrichment of a cardiac stem/progenitor cell population. Among potential candidate markers for cardiac progenitors is platelet-derived growth factor receptor-alpha (Pdgfra); a cell-surface protein expressed on cardiac progenitor cells [5, 6]. Differentiation of mouse ESCs into cardiomyocyte reveals that this sub-population co-expresses PDGFRA and another marker FLK1 [7].

Initially thought to be unipotent, germline stem cells (GSCs) cultured under defined conditions are capable of acquiring pluripotency [8–13]. Recently, we demonstrated that multipotent germline stem cells (mGSCs) express markers of pluripotency, can differentiate into derivatives of all three germ layers *in vitro*, and are capable of forming teratomas in immune deficient mice [14]. Additionally, mGSCs are capable of differentiatiate into cardiomyocytes and endothelial cells *in vitro*, and *in vivo* studies suggest that these cells have ability to restore functions in damaged hearts of animal models [5, 15].

In this study, we used defined culture conditions to derive cardiac stem/progenitor cells from mouse mGSCs. Particularly, we found that isolation of PDGFRA expressing cardiac stem/progenitor cells were capable of effective differentiation into cardiomyocytes *in vitro*, and displayed *in vivo* functional properties when transplanted in the hearts of a rat model of myocardial infarction. Together these findings suggest that mGSCs are a potential stem cell source from which to derive cardiac stem/progenitor cells capable of repairing damaged myocardial tissue.

## RESULTS

### Effects of differentiation medium on mGSCs cardiac induction

Our first steps were to determine the optimal culture conditions that promote cardiac differentiation of mGSCs. As such, embryoid bodies (EBs) derived from mGSCs were cultured for 3 days in either IMDM/FBS, KO-DMEM/KSR, KO-DMEM/FBS, or N2/B27 medium. To evaluate the temporal changes in gene expression associated with early cardiogenesis, we assessed the expression of *Brachyury*, a T-box domain-containing transcription factor expressed in embryonic mesoderm that is down-regulated following initiation of tissue-specific patterning [16]. Exposure of mGSC-derived EBs to N2/B27 medium was associated with a marked increase in *Brachyury* gene expression (Supplementary Figure 1). This up regulation is consistent with previous findings showing that EBs display a characteristic spike in *Brachyury* expression at the onset of cardiac differentiation [16].

### Analysis of FLK1 and PDGFRA expression during differentiation

We next evaluated cardiac differentiation of mGSC-derived EBs following exposure to N2/B27 culture medium (without growth factors) by using flow cytometry to assess PDGFRA and FLK1 expressing populations. Following exposure to N2/B27 culture medium (without growth factors), we observed the fraction of PDGFRA^+^ cells increase by 0.1%, 9.6%, and 13.3% after 3, 4, and 5 days, respectively. In contrast, FLK1^+^ expressing cells accounted for only 0.2%, 0.5%, and 1.0% of this same population (Supplementary Figure 2A). Culture of mGSC-derived EBs in MEMα containing 10% FBS promoted a 1.3%, 7.9%, and 13.8% increase in FLK1+ expressing cells after 3, 4, and 5 days, but was conversely associated with only a small fraction of PDGFRA+ cells (Supplementary Figure 2B).

### Analysis of cardiac lineage differentiation potential of PDGFRA^+^ population

**Figure 1:**
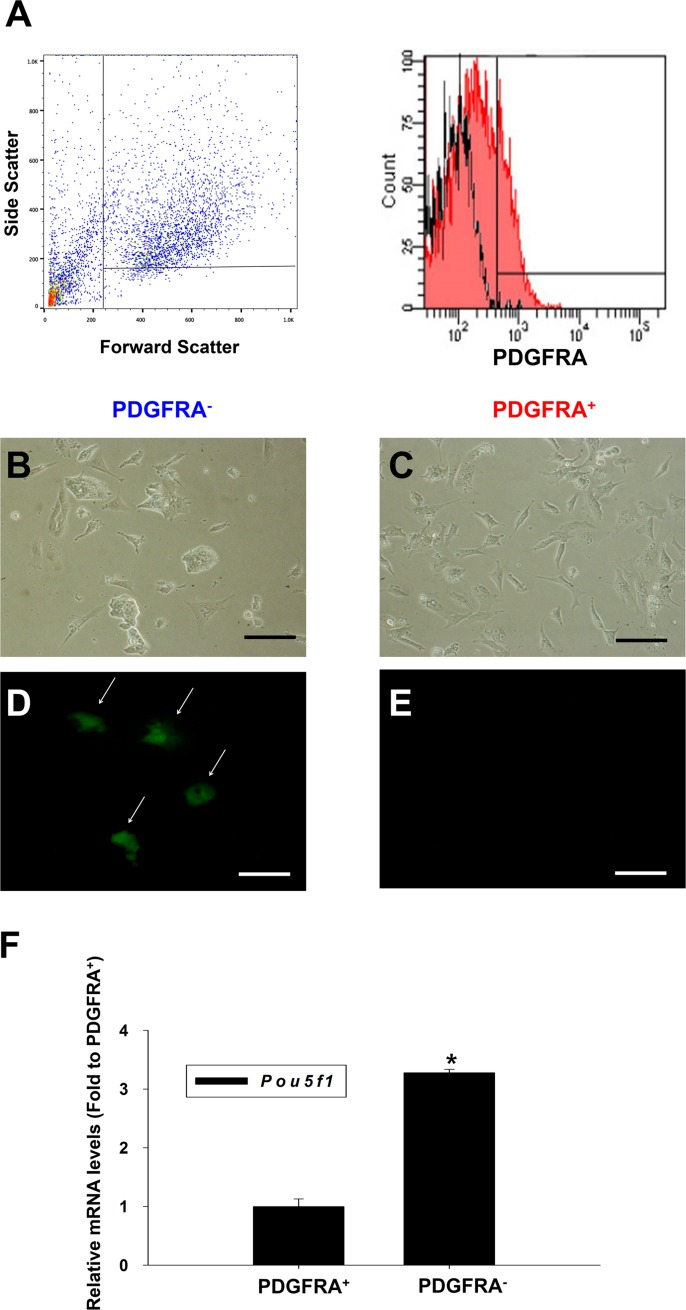
Characterization of PDGFRA^+^ and PDGFRA^−^ sorted cell population **A**. Flow cytometric analysis of the PDGFRA expression in differentiating mGSCs. **B**.-**E**. Images on day 2 after plating of mGSC-derived PDGFRA^+^ and PDGFRA^−^ cells. **B**., **D**. PDGFRA^−^ sorted cells **C**., **E**. PDGFRA^+^ sorted cells. **B**., **C**. phase contrast, and **D**., **E**. fluorescent imaging showing POU5F1 expression. **F**. Quantification of *Pou5f1* gene expressions. The *Pou5f1* gene expression levels normalized to that of PDGFRA^+^ cells (mean ± SEM; *n* = 3). Means with different letters are significantly different (*P* < 0.05). B-E: Scale bar = 100 μm.

After 5 days of culturing mGSCs in N2/B27 culture medium, the cells were FACS sorted by gating for PDGFRA^+^ or PDGFRA^−^ cell populations (Figure 1A). These respective cell populations were then collected and plated on 0.1% gelatin-coated 24-well culture dishes in N2/B27 medium containing 30 ng/mL bFGF and 10 ng/mL VEGF. Two days after plating, the expression of *Pou5f1,* a marker of pluripotency was assessed. Specifically, the mGSCs used in these experiments were derived from transgenic mice expressing Enhanced Green Fluorescent Protein (EGFP) under the control of the *Pou5f1* promoter and distal enhancer elements. Whereas POU5f1 mediated EGFP expression was not observed in PDGFRA^+^ cells, PDGFRA^−^ derivatives showed robust EGFP expression. This suggests that undifferentiated mGSCs are contained within the PDGFRA^−^ population (Figure 1B-1E). Further analysis *of Pou5f1* gene expression corroborated this finding, as *Pou5f1* transcript levels were significantly lower (*P* < 0.05) in PDGFRA^+^ cells compared to PDGFRA^−^ cells (Figure 1F).

**Figure 2:**
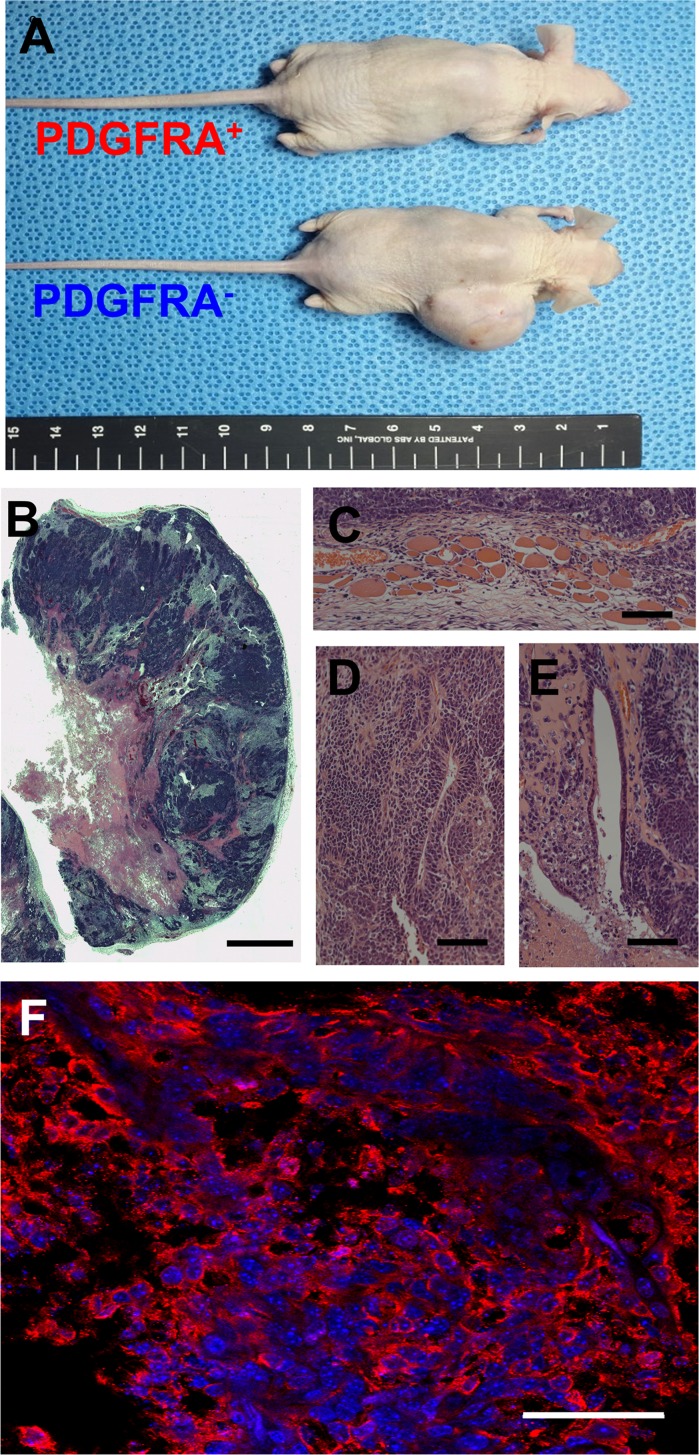
Teratoma assay of PDGFRA ^+^ and PDGFRA^−^ cells **A**. Teratoma from PDGFRA^−^ cells in nude mice. Presence of all three primary germ-layer derivatives in a teratoma (**B**. image of H&E stained teratoma, **C**. Muscle; mesoderm, **D**. Neural tissue; ectoderm, **E**. Endothelium; endoderm). **F**. Fluorescence microscopy image of a teratoma immunostained for KI67. (**B**. Scale bar = 2.5 mm, **C**.-**E**. Scale bar = 100 μm, **F**. Scale bar = 50 μm)

Suspecting that an undifferentiated mGSC population was contained within the PDGFRA^−^ population, we subcutaneously transplanted sorted PDGFRA^+^ and PDGFRA^−^ cells into mice. Within 4 months, Ki67^+^ teratomas were observed in all mice transplanted with PDGFRA^−^ cells (Figure 2A-2F). This suggests that pluripotent characteristics retained by PDGFRA^−^ cells derived from mGSCs are not immediately amendable for use in cardiac cell therapy. In contrast, mice transplanted with PDGFRA^+^ cells did not form teratomas, even as far out as 8 months post-implantation (Figure 2A).

**Figure 3:**
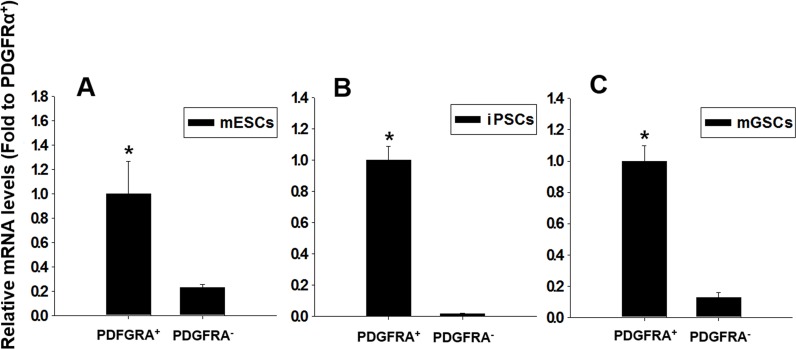
Relative expressions of cardiac-specific gene *cTnT*, in PDGFRA^+^ and PDGFRA^−^ cell populations **A**. mESCs, **B**. iPSCs, **C**. mGSCs. qRT-PCR analysis of FACS sorted PDGFRA^+^ cells for the cardiac-specific marker gene, *cTnT*. The expression levels are normalized to those of PDGFRA ^+^ (mean ± SEM; *n* = 3). Means with different letters are significantly different (*P* < 0.05).

We next investigated whether PDGFRA^+^ cells were capable of efficient cardiomyogenesis. *In vitro* cultures of mGSCs along with control cultures of mESCs and iPSCs, were maintained in N2/B27 differentiating medium for 5 days, following which PDGFRA^+^ and PDGFRA^−^ populations were sorted, collected, and re-plated in the presence of N2/B27 differentiating medium for an additional 5 days. The collected cells were analyzed for the expression of *cTnT*, a transcription factor associated with cardiomyocytes differentiation. Unlike PDGFRA^−^ cell populations, PDGFRA^+^ cells derived from mGSCs, mESC, or iPSCs were associated with a significant up-regulation of *cTnT* gene expression (Figure 3). This suggests that compared to PDGFRA^−^ cells, PDGFRA^+^ cells have greater cardiac stem/progenitor-like potential, and more capable of differentiating into cardiomyocytes.

### Effect of BMP4 and FBS on derivation PDGFRA^+^ population

**Figure 4:**
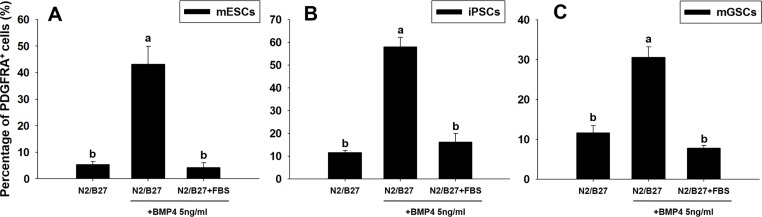
Changes in PDGFRA population in PSCs following BMP4 and FBS-induced differentiation The percentage of PDGFRA^+^ cells was evaluated in **A**. mESCs, **B**. iPSCs, and **C**. mGSCs, following exposure to N2/B27 medium supplemented with BMP4 (5 ng/ml) or FBS (15% final concentration). Means with different letters are significantly different (*P* < 0.05).

The BMP signaling pathways play a pivotal role in cardiogenesis [17, 18], and our previous findings show that the culture of mGSCs with BMP4 (under serum-free conditions) promotes cardiomyocyte differentiation [14]. To investigate the influence of BMP4 and FBS to induce PDGFRA^+^ cardiac progenitor differentiation, mGSCs were seeded in 24-well ultra-low attachment plate at a density of 4 ×10^4^ cells/well, and treated with N2/B27 medium containing BMP4 (5 ng/mL) and/or FBS (15%). Parallel control experiments used mESCs and iPSCs. Five days later, the proportion of PDGFRA expressing cells contained within EBs was assessed by flow cytometry. For cells cultured in N2/B27 medium alone, the percentage of PDGFRA^+^ cells contained within EBs derived from mESCs, iPSCs, or mGSCs, was 5.3 ± 1.2%, 11.6 ± 0.9%, and 11.6 ± 2.7%, respectively (Figure 4; mean ± SEM; *n* = 3). In contrast, for EBs exposed to N2/B27 medium supplemented with BMP4, the percentage of PDGFRA^+^ cells derived from mESCs, iPSCs, and mGSCs was significantly increased (43.1 ± 6.8%, 58.0 ± 4.2%, and 30.6 ± 2.7%, respectively; mean ± SEM; *n* = 3) (Figure 4). Following exposure to N2/B27 medium supplemented with both BMP4 and 15% FBS, the PDGFRA^+^ population contained within EBs derived from mESCs, iPSCs, or mGSCs cultures was 4.2 ± 1.9%, 16.2 ± 3.8%, and 7.8 ± 0.7%, respectively (Figure 4; mean ± SEM; *n* = 3). Exposure of EBs to MEMa medium containing BMP4 similarly increased the proportion of FLK1^+^ expressing cells in a dose-dependent manner (Supplementary Figure S3). The respective increases to either a PDGFRA+ or FLK1^+^ cell population suggests that BMP4 functions to promote the expansion of cardiac progenitors. In contrast, the presence of serum appears to inhibit this process.

### *PdgfR-a* signaling is critical for cardiac differentiation of PSCs

**Figure 5:**
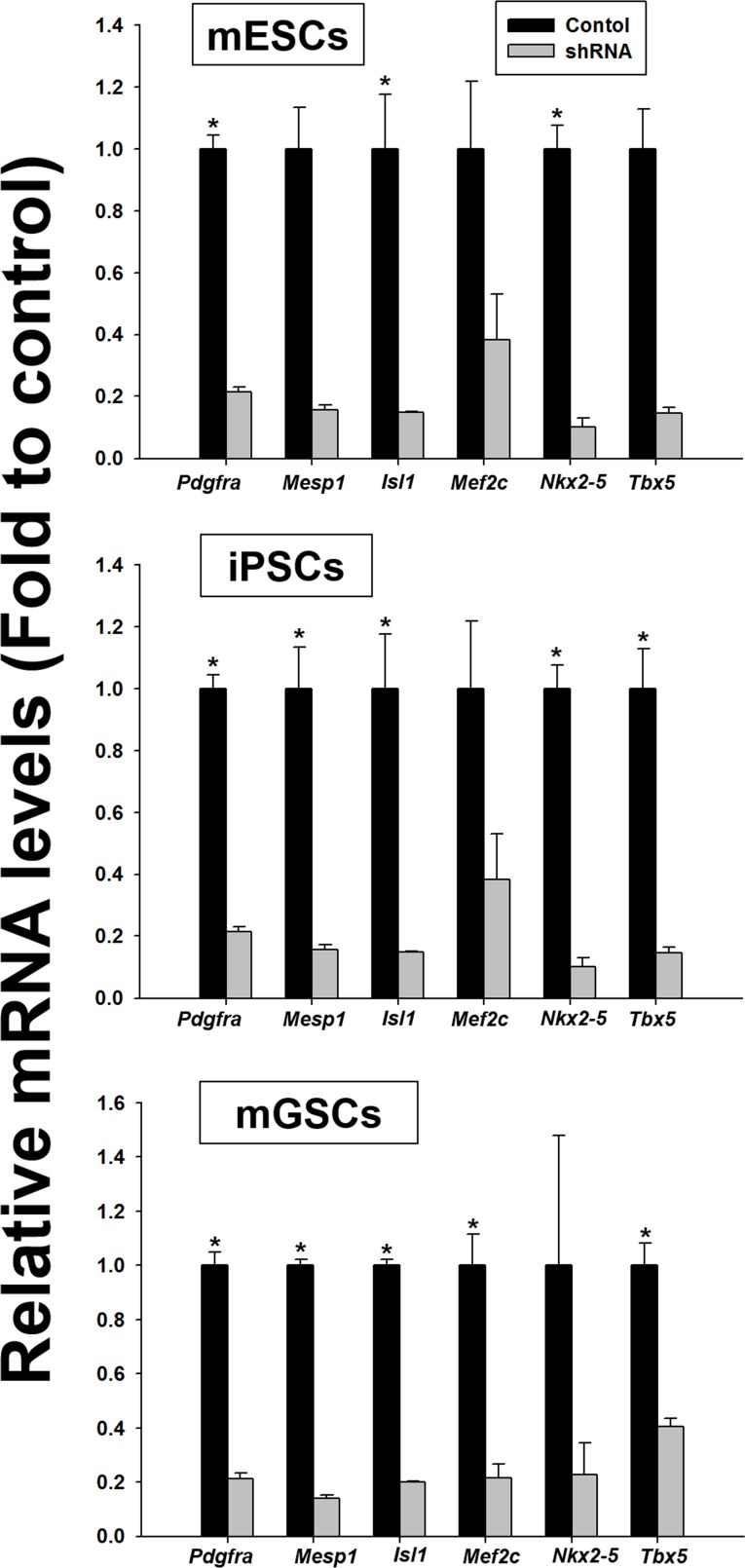
Comparison of the relative expression of cardiac lineage-specific genes in the *shPdgfra* transfected PSCs PSCs were transfected with empty vector control or sh*PdgR-α* and selected with puromycin (0.2 μg/mL). Cardiac differentiation in N2/B27 medium was evaluated in presence of 5 ng/mL of BMP4 (mean ± SEM; *n* = 3). Means with different letters are significantly different (*P* < 0.05).

To further investigate the *in vitro* dependency of mGSCs on *Pdgfra* signaling for cardiac differentiation, we used shRNA to knockdown *Pdgfra* expression (sh*Pdgfra*). Parallel control experiments used mESCs and iPSCs. Stable shRNA-mediated *Pdgfra* knockdown was confirmed by qRT-PCR (Figure 5). After 5 days in the EB-inducing culture medium, either empty vector control or sh*Pdgfra* cell populations were evaluated for the expression levels of cardiac lineage-specific markers (i.e. *Mesp1*, *Isl1*, *Mef2c*, *Nkx2-5*, and *Tbx5*; Figure 5). Results show that following *Pdgfra* knockdown, there is a substantial reduction of the cardiac lineage-specific gene expression levels in EBs derived from mESC, iPSC, and mGSC cell types. These findings suggest that activated *Pdgfra* signaling is necessary for the expression of cardiac-related gene expression during cardiac differentiation.

### Effect of growth factors in *PdgfR-a* expression during cardiac differentiation of PSCs

**Figure 6:**
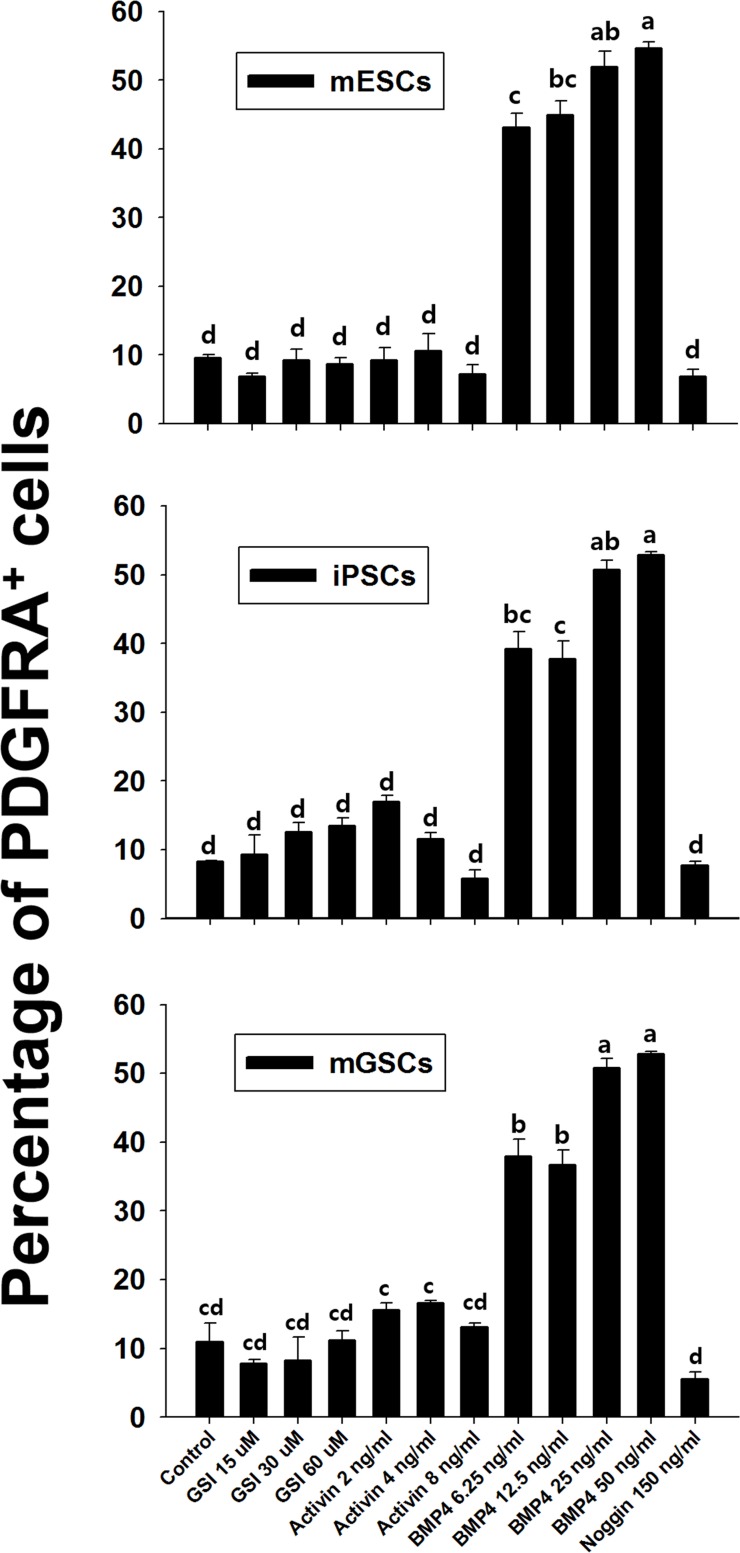
Effect of growth factors on *Pdgfra* expression Flow cytometric analysis of anti-Pdgfra antibody selected cells. The vertical bar graph shows comparison of fluorescence of Pdgfra selected cells treated with PE-conjugated antibody and that of isotype control (mean ± SEM; *n* = 3). Means with different letters are significantly different (P < 0.05).

We next sought to determine whether the proportion of *Pdgfra*-expressing cardiac stem/progenitor cells derived from mGSCs could be influenced by additional growth factors. mGSCs, along with pluripotent control cultures of ESCs and iPSCs, were maintained in N2/B27 medium and were subsequently treated with various concentrations of factors known to promote cardiac differentiation. These included: gamma-secretase inhibitor (GSI), activin, BMP4, and Noggin [19–21]. Compared to untreated cells cultured in N2/B27 medium alone, the exposure of mGSCs, ESCs, or iPSCs, to BMP4 (6.25 ng/mL) showed significant increases in the proportion of PDGFRA expressing cells (mGSC = 11.0 ± 2.7% *vs*. 37.9 ± 2.5%; ESC = 9.6 ± 0.5% *vs*. 43.2 ± 2.0%; iPSCs = 8.2 ± 0.2% *vs*. 39.2 ± 2.5%; *P* < 0.05). Higher concentrations of BMP4 resulted in further increases to the proportion of PDGFRA expressing cells (Figure 6). In contrast, no significant increases to the number of PDGFRA^+^ cells was observed among mGSCs cells treated with GSI, activin, or Noggin (Figure 6). Similar results observed with control mESCs and iPSCs further substantiated these finding, and suggested that the expansion of a PDGFRA expressing population is strongly influenced by BMP4 signaling.

**Figure 7:**
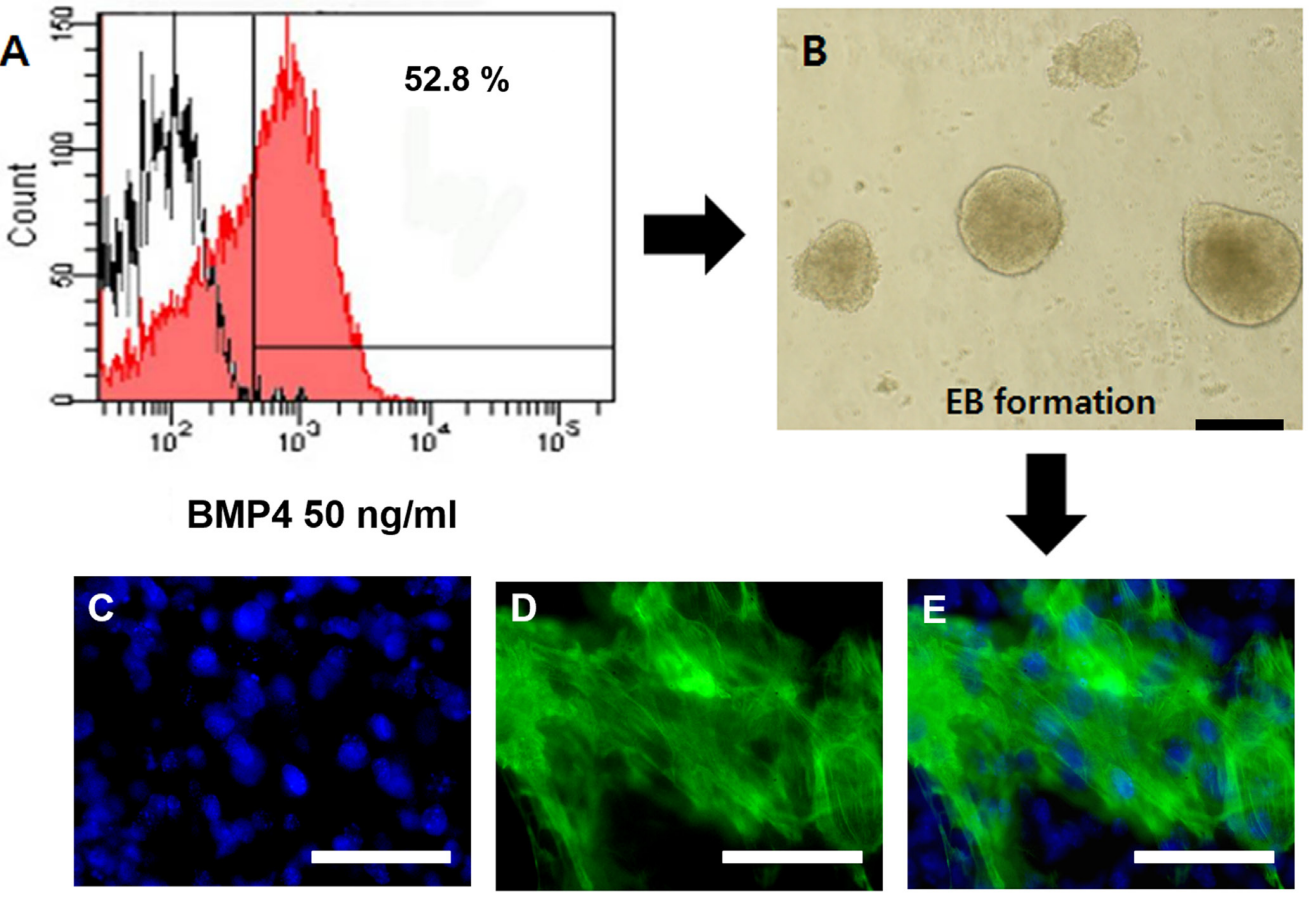
Expression of *cTnT* in PDGFRA^+^ derived cardiomyocytes **A**. Expression of PDGFRA in mGSC-derived EBs. **B**. PDGFRA^+^ cells were sorted by flow cytometry and the generated EBs at day 2, the EBs were re-plated on 0.1% gelatin-coated coverslip for additional 4 days. **C**.-**E**. The expression of functional cardiomyocyte marker protein, *cTnT*, was detected by immunocytochemistry. (Scale bar = 100 μm).

To evaluate the cardiogenic potential of mGSC-derived EBs, we expanded the number of PDGFRA^+^ cells by exposing cells to BMP4 (50 ng/mL) (Figure 7A). The expanded PDGFRA^+^ cell population was subsequently enriched by cell sorting, and the cells seeded on 96-well ultra-low attachment plates at a density of 5 × 10^3^ cells/well in N2/B27 medium containing 10 ng/mL VEGF and 30ng/mL bFGF. In order to establish monolayers of differentiating cardiomyocytes, the 2-day culture of re-aggregated EBs (Figure 7B) were re-plated on 0.1% gelatin-coated tissue culture plates. After another 4 days in culture, the BMP4-induced PDGFRA^+^ population were found to robustly express the cardiomyocytes marker, cTnT (Figure 7C-7E). There was no statistically significant difference in the expression of *cTnT* across either PDGFRα^+^ or FLK1^+^ populations derived from mGSCs (Supplementary Figure S4). Also, spontaneous beating was observed as day 5 post plating (S1 Video). These findings suggest that PDGFRA^+^ and FLK1^+^ cell population may have similar potential to undergo cardiomyocyte differentiation.

### Therapeutic regeneration for damaged heart by transplanting PDGFRA^+^ cardiac progenitors

**Figure 8:**
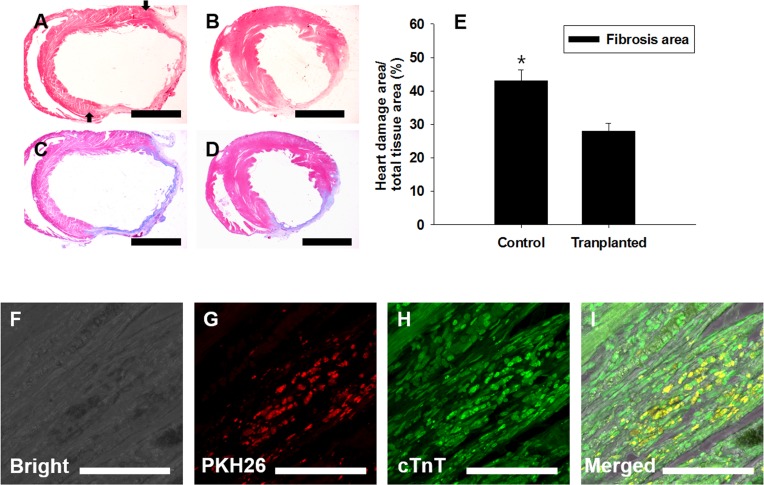
Transplantation of PDGFRA^+^ population in a rat MI model FACS-sorted PDGFRA^+^ cells were transplanted into MI-induced rat hearts. Four weeks after transplantation, Mason-trichrome (MT) staining and immunocytochemical staining for cardiomyocyte marker, *cTnT*, were performed to assess the functional activity of transplanted PDGFRA^+^ cells. **A**., **C**. PBS-injected control, **B**., **D**. MI heart injected with PDGFRA^+^ cells derived from mGSC culture, **A**., **B**. H&E staining, **C**., **D**. MT staining to detect the heart with fibrosis. **E**. Quantification of fibrotic area (mean ± SEM; *n* = 3), **F**.-**I**. Immunocytochemical analysis with *cTnT*, **F**. Bright field, **G**. red fluorescence in PKH26-labeled cells, **H**. PDGFRA derived cells, **I**. Merged images of **F**., **G**., **H**. (Scale bar in A-D = 5 mm; F-I = 100 μm).

The therapeutic efficacy of the PDGFRA^+^ cells generated from mGSC-derived EBs was evaluated using an *in vivo* rat MI model. Following a 5-day induction with BMP4 (50 ng/mL), the PDGFRA^+^ population was enriched by cell sorting and subsequently injected into peri-infarct zones of the infarcted rat hearts. After 4 weeks, the fibrotic area of infarcted hearts was evaluated by Mason Trichrome staining (Figure 8A-8D). Compared to the hearts of control rats administered with PBS, quantification of fibrotic regions in the hearts of rats that were transplanted with PDGFRA^+^ cells showed significantly smaller areas of fibrosis, (Figure 8E).

To further evaluate the ability for PDGFRA^+^ cells to engraft within recipient heart tissue, donor cells were labeled with PKH26 dye for *in vivo* tracking. Under fluorescence microscopy, we observed numerous PKH26-labeled cells that co-expressed cTnT. These findings provide strong evidence that the PDGFRA^+^ cells functionally differentiated into cardiomyocytes (Figure 8F-8I).

## DISCUSSION

To advance cardiac stem cell therapy, and provide effective repair of damaged heart tissue, the ideal candidate cell should share characteristics with cardiac stem/progenitor cells and be capable of functionally differentiating into all cardiac-specific lineages. Using the ligand BMP4, we demonstrate that cardiomyocytes can be efficiently generated by expanding a PDGFRA expressing cell population derived from mouse mGSCs. Moreover, we show that this PDGFRA expressing cell population has the potential to promote cardiac regeneration *in vivo*. Notably, we observed that PDGFRA^+^ cells have a greater potential to differentiate into cardiomyocytes *in vitro* than their PDGFRA^−^ cells. Rather, PDGFRA^−^ cells derived from mGSCs readily formed teratomas in mice, indicating that this cell population harbors unwanted pluripotency that would have dire implications if introduced into the clinic [22, 23].

Several reports suggest that the expression of *Pdgfra* is associated with the cardiomyocyte lineage, as its expression is observed not only in the developing mesoderm at embryonic d7.5, but is also expressed by pluripotent stem cells undergoing cardiac differentiation *in vitro* [6, 7, 24]. We found that *Pdgfra* is necessary for cardiac-specific differentiation, as abrogation of *Pdgfra* expression using shRNA dramatically reduced the expression of genes associated with cardiac-specific differentiation. Particularly, sh*Pdgfra* knockdown resulted in a significant reduction in *Isl1* expression in mGSCs cultures. As a LIM homeodomain transcription factor, *Isl1* expression is required within a subset of undifferentiated cardiac progenitors for the development of cardiomyocytes [25]. These findings provide further support for *Pdgfra* signaling as having a critical role for in cardiac cell differentiation.

*Flk1* is an early mesodermal surface marker that plays a central role not only in hematopoietic and endothelial differentiation but also in cardiomyocyte generation [26, 27]. In the current study, we observed that cardiac progenitors derived from either FLK1^+^ or PDGFRA^+^ cell populations were each capable of undergoing cardiomyocyte differentiation *in vitro*; however, the expansion of PDGFRA^+^ and FLK1^+^ cell populations each required different culture medium and appeared to respond differently to serum. Particularly, under a serum-free differentiation system, there was very little expansion of FLK1^+^ cells, but a marked increase in the PDGFRA expressing population. Conversely, in serum-containing MEMα culture medium, there was an increase in the FLK1^+^ population but not in the PDGFRA^+^ cell population. This discrepancy may be explained by the inherent complexity of a serum, which limits our ability to completely understand the key factors and mechanisms that regulate the differentiation processes. Subsequent experiments aim to further elucidate the difference between PDGFRA^+^ and FLK1^+^ cells, and characterize their respective potential to undergo cardiomyocyte differentiation, both *in vitro* and *in vivo*.

Several growth factors, in addition to BMP4, have also been implicated in promoting the cardiac differentiation of pluripotent stem cells [9, 17, 18, 28–35]. Particularly, inactivation of Notch signaling by treatment with GSI, promotes ESC differentiation into cardiac mesodermal cells [19]. GSI effectively blocks signaling pathways by preventing the cleavage of the intracellular fragment of the Notch receptor. In our study, we observed that use of GSI in the culture system had a limited impact on promoting the expansion of the PDGFRA^+^ cell population. Similarly, the sequential treatment of hESCs with activin A and BMP4 for 5 days is associated with improving the overall efficiency of cells undergoing cardiac differentiation [36]. That said, the generation of cardiac progenitor cells from hESC and hiPSC cultures can occur in the absence of activin. In this study we observed that activin had little to no impact on promoting increased expansion of the PDGFRA expressing population.

Here we report on the derivation of cardiac progenitor-like cells from mGSCs by specifically enriching for a PDGFRA expressing cell population. Importantly, we show that in a rat MI model, transplanted PDGFRA^+^ cells derived from mGSCs were capable of differentiate into cardiomyocytes and significantly reduced areas of fibrotic and damaged heart tissue. Our findings open the door for cardiac progenitors derived from mGSCs, and potentially circumvent ethical quandaries surrounding ESCs, as well as the challenges associated with non-autologous stem cell transplantation. Additionally, there are many gender-specific differences in cardiac diseases that have yet to be clarified [37]. Subsequent use of mGSC-based cell therapy for cardiac regeneration may also provide an alternative approach to addressing gender-specific cardiac pathology.

## MATERIALS AND METHODS

All procedures were performed according to guidelines for the ethical treatment of animals and approved by Institutional Animal Care and Use Committee in Chung-Ang University, Seoul, Korea.

### Cell culture and differentiation

mGSCs were derived from transgenic mice expressing Enhanced Green Fluorescent Protein (EGFP) under the control of the POU5f1, promoter and distal enhancer. This mouse represents an effective tool for studying or monitoring pluripotency since Pouf1 is a robust marker for pluripotency [14]. The E14 mouse ES cell line, neural stem cell-derived iPS (NSC-iPS; iPS) cell line, and mGSCs were cultivated on mouse embryonic fibroblasts (MEFs) in standard ESC medium Dulbecco's modified Eagle's medium (DMEM) supplemented with 15% FBS, 1×MEM nonessential amino acids, 2 mM L-glutamine, penicillin/streptomycin, 50 μM β-mercaptoethanol, and 10^3^ U/mL leukemia inhibitory factor (LIF) as described previously described [14]. Forty minutes prior to differentiation, PSCs were plated on gelatin-coated dishes containing ESC media to remove the feeder cells. Subsequently, PSCs were transferred to 24-well ultra-low-attachment plates (Costar), and cultured with 4 ×10^4^ cells/mL/well of the following media: standard ESC medium, IMDM/FBS medium [IMDM supplemented with 15% FBS, 1×MEM nonessential amino acids, 2 mM L-glutamine, 50 μM β-mercaptoethanol, Knock-out DMEM/B27 medium (KO-DMEM [Invitrogen] supplemented with 2% B27) KO-DMEM/KSR medium (KO-DMEM supplemented with KO serum replacement [KSR; 15%], 1×MEM nonessential amino acids, 2 mM L-glutamine, 50 μM β-mercaptoethanol), KO-DMEM/FBS (KO-DMEM supplemented with 15% FBS, 1×MEM nonessential amino acids, 2 mM L-glutamine, 50 μM β-mercaptoethanol), or N2/B27 medium (DMEM-F12 supplemented with 1% B27, 0.5% N2, 100 μM β-mercaptoethanol, 2 mM L-glutamine). Additional growth factors (e.g. BMP-4) were supplemented to the media as indicated. For induction of FLK1 expression, mGSCs were differentiated as previously described. Single cell dispersions of mMGSCs were briefly seeded on gelatin coated 6-well tissue culture plates in the presence of MEMα/FBS medium (MEMα supplemented with 10% FBS, 50 μM β-mercaptoethanol) as previously described [36].

### *PdgfR-a* knockdown

PSCs were seeded at a density of 5 ×10^4^ cells/well in 24-well plates and were incubated for 24 hours. The cells were then transfected with a single short hairpin RNA [shRNA; Empty Vector Control (TRC pLKO; GE Healthcare Dharmacon, Lafayette, CO) or Sh*Pdgfra* (TRCN0000001423; E Healthcare Dharmacon, Lafayette, CO)] vector construct targeting *Pdgfra* expression. According to the manufacturer's instructions, transfected cells were cultured in puromycin (0.2 μg/mL; Sigma, St. Louis, MO) for approximately two weeks in order to allow stable selection.

### Real-time quantitative reverse transcriptase-polymerase chain reaction (qRT-PCR)

Total RNA was isolated and prepared from cells using the PureLink RNA Mini Kit (Invitrogen). RNA was reverse transcribed using the Superscript III Reverse Transcriptase (Invitrogen) according to the manufacturer's instructions. qRT-PCR was performed using a 7500 Real-Time PCR System (Applied Biosystems, Carlsbad, CA) and the synthesized cDNA was amplified using TaqMan Gene Expression Master Mix (Applied Biosystems). All gene expression levels were normalized to levels of GAPDH. All TaqMan primers and probes used were commercially obtained from Applied Biosystems.

### Flow cytometry and cell sorting

Dissociated cells were stained with the following antibodies purchased from eBioscience: conjugated anti-mouse PDGFRA (CD140a), phycoerythrin (PE), conjugated anti-mouse FLK1-Allophycocyanin (APC), rat igG2a isotype control-APC, and rat igG2a isotype control-PE. The dissociated cells were suspended in phosphate-buffered saline (PBS) supplemented with 1% FBS, 10 mM [4-(2-hydroxyethyl)-1-piperazineethanesulfonic acid] HEPES, 1 mM pyruvate, antibiotics (50 U/mL penicillin and 50 μg/mL streptomycin), 1 mg/mL glucose (PBS-S). The cells were then incubated with the appropriate antibodies for 20 minutes on an ice bath and washed twice with excess PBS-S for FACS analysis. After the final wash, the cells were resuspended (1 ×10^6^ cells/mL) in PBS-S containing 1 μg/mL propidium iodide (PI; Sigma) and kept in dark on an ice bath until further analysis. Flow cytometric analyses and cell sorting were performed using the Dual-Laser FACS Aria II (BD Biosciences, Center for Research Facilities, Chung-Ang University). The sorted cells were centrifuged and plated in 0.1% gelatin-coated coverslip or V-shaped ultra-low attachment 96-well plates (Corning) at a density of 5 × 10^3^ cells/well in N2/B27 supplemented medium (Invitrogen) containing 10 ng/mL of vascular endothelial growth factor (VEGF; R&D Systems, Minneapolis, MN) and 30 ng/mL of basic fibroblast growth factor (bFGF; BD Biosciences, San Jose, CA). Also, PDGFRA positive and negative cells were sorted by FACS and were injected into the flank of athymic nude mice in 100 μl phosphate-buffered saline (PBS). Four to six months after the injection, teratomas were collected for H&E staining.

### Immunohischemistry and immunocytochemistry

To characterize protein expression of cells using immunocytochemical analysis, cells were fixed with 4% paraformaldehyde for 30 minutes at room temperature, permeabilized with 0.1% Triton X-100 (Sigma) for 15 minutes, and then incubated with 5% (w/v) bovine serum albumin (BSA; Roche, Basel, Switzerland) at room temperature for 30 minutes. Subsequently, the cells were incubated with primary antibody cTnT (Thermo Scientific, Logan, UT), were diluted 1:200 with a solution of 5% BSA, and were incubated overnight at 4°C. After two washes with PBS, the cells were incubated with the secondary AlexaFluor-488 goat anti-mouse antibody (Invitrogen) and were diluted 1:200 with 5% BSA for 1 hour at room temperature in dark. Finally, the cells were washed twice with PBS and mounted on glass slides with the Vectashield media containing 4′, 6-diamidino-2-phenylindole (DAPI) stain (Vector Laboratories, Burlingame, CA).

### Rat MI models

An MI model was performed as previously described [38]. In brief, Sprague-Dawley rats (age: 7-9 weeks; Koatech, Pyeongtaek, Korea) were anesthetized with ketamine (350 mg/kg) by intraperitoneal (i.p.) injection. Rats were endotracheally intubated using polyethylene (size 90) tubing and were provided with positive-pressure ventilation of oxygen-supplemented room air by a small animal volume-controlled ventilator. A left thoracotomy was performed between the fourth intercostals space, and the pericardium was opened. An MI was induced by ligating the left anterior descending (LAD) coronary artery with 6-0 nylon suture. The rats were randomly assigned into two groups: a PDGFRA^+^-injected treatment group and a PBS-injected control group. To detect donor cells injected into peri-infarct area of the rat heart, the PDGFRA^+^ cells were pre-stained with PKH26 dye before transplantation. Using a 31-gauge Hamilton syringe, 1 × 10^5^/100 μL of PDGFRA sorted cells were equidistantly transplanted into three different peri-infarct zones of the heart. To avoid immune-rejection, rats were injected (i.p.) daily with cyclosporine (10 mg/mL; Chong Kun Dang Pham, Seoul, Korea) during the first week, followed by a lower daily injection of 5 mg/mL thereafter until the appropriate time for analysis. Four weeks after transplantation, the rats were euthanized and their hearts were collected. The excised hearts were retrograde perfused with ice-cold PBS to wash the coronary vasculature, then were fixed overnight at 4°C with 4% paraformaldehyde, followed by a second overnight incubation in 15% sucrose at 4°C. The heart tissue was embedded in paraffin or frozen in optimal cutting temperature (OCT) compound (Cellpath, Wales, UK). Sections (7-μm) were stained by Mason's trichrome (MT) to determine the infarct size. Areas of infarction were quantified by measuring the area of fibrosis, shown as blue-stained collagen fibers. MT-stained images were taken per tissue section with the Nikon TE2000 microscope accompanied with NIS Elements imaging software (Nikon), and infarcted regions per section were measured using image J.

### Statistical analysis

Statistical analysis was conducted using SPSS software (version 18; SPSS Inc., Mechanicsburg, PA). Analysis of variance (ANOVA), Tukey's honestly significant difference (HSD) test, and independent *t*-test were used to assess significant differences. P-values under 0.05 were considered to be statistically significant.

## SUPPLEMENTARY MATERIALS FIGURES




