# Supplementary material for: Platelet-derived growth factor receptor-alpha positive cardiac progenitor cells derived from multipotent germline stem cells are capable of cardiomyogenesis in vitro and in vivo
Source: Oncotarget. 2017 Mar 31;8(18):29643–56. doi: 10.18632/oncotarget.16772 (PMC5444692; doi:10.18632/oncotarget.16772)
Supplement: Supplementary file 1 [file oncotarget-08-29643-s001.pdf]

Platelet-derived growth factor receptor-alpha positive cardiac progenitor cells derived from multipotent germline stem cells are capable of cardiomyogenesis *in vitro* and *in vivo*

Supplementary Material

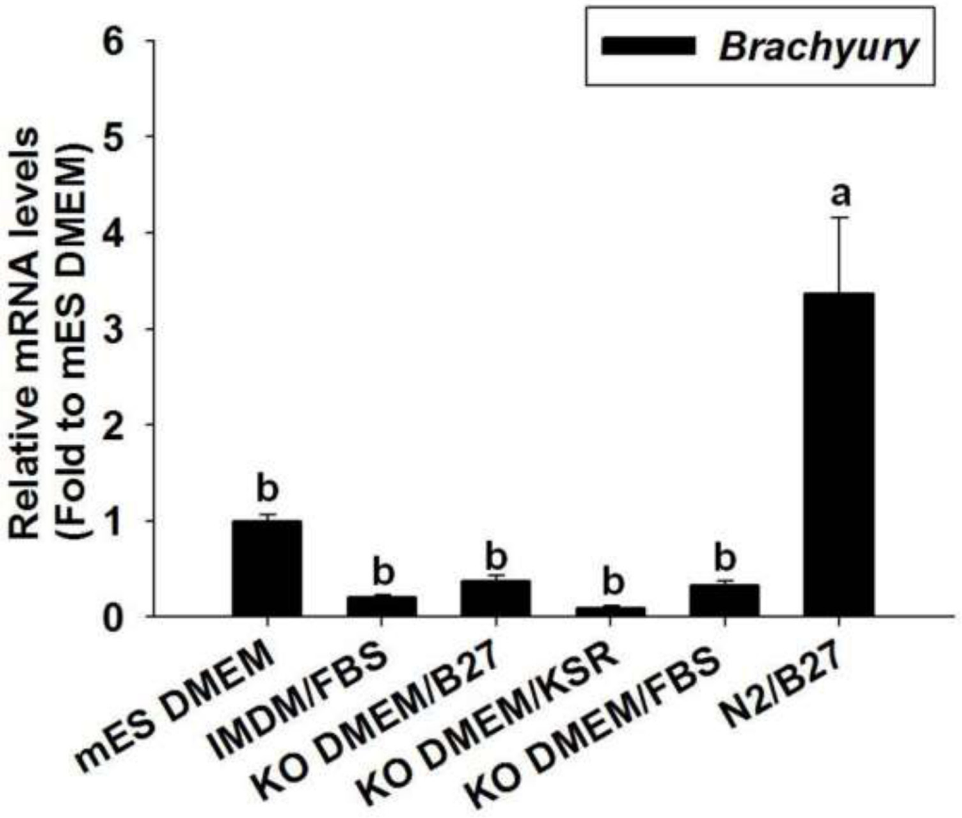

Supplementary Figure 1: Screening of cardiac differentiation medium. The expression of *Brachyury* was evaluated by qRT-PCR following exposure of cells to various differentiating culture mediums for 3 days after differentiation. Values are means ± SEM (n = 3). Bars within a group indicated with different letters are significantly different ($P < 0.05$).

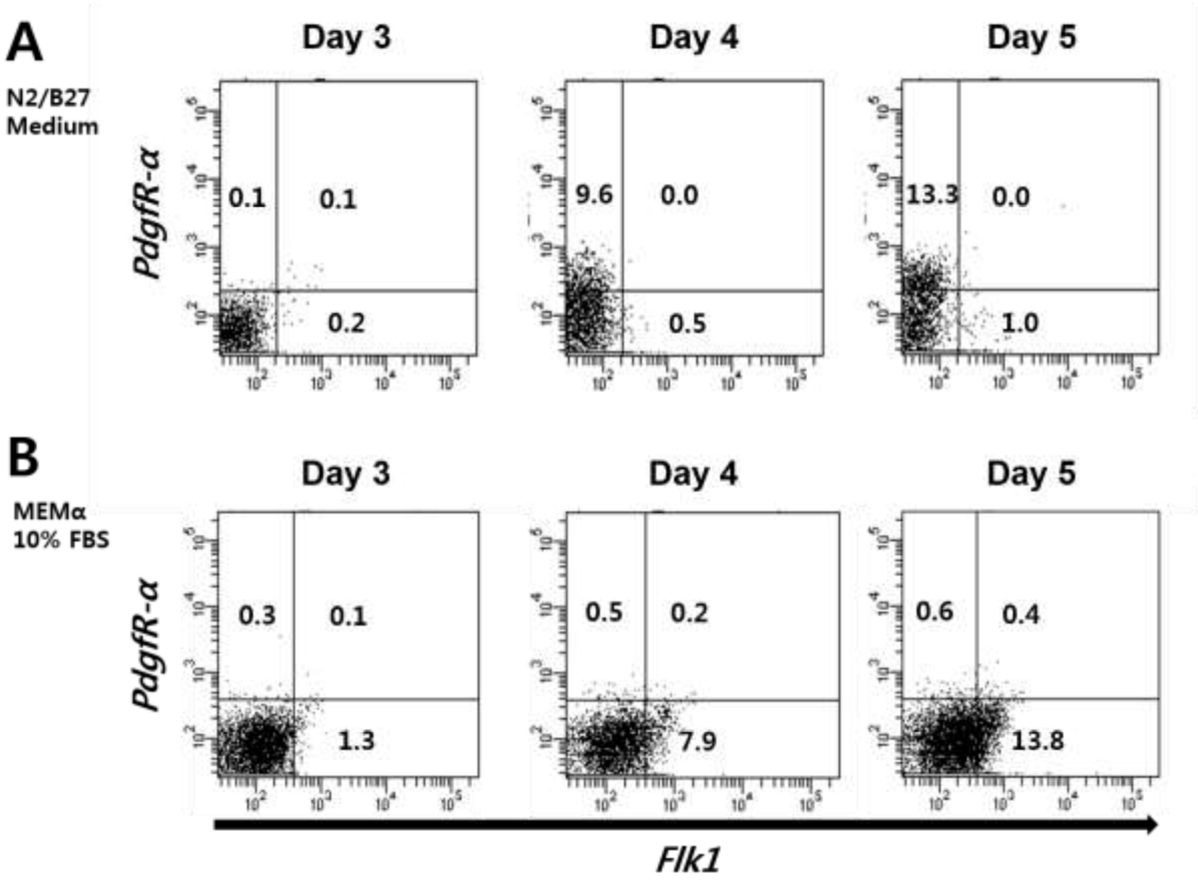

Supplementary Figure 2: PDGFR-α and FLK1 expression in differentiating mGSCs. A. mGSCs were seeded in ultralow attachment plates containing N2B27 medium. On days 3-5 after differentiation, the EBs were dissociated and stained with PE-conjugated anti-PDGFR-*α* and anti-FLK1 antibody. **B.** mGSCs were seeded in gelatin-coated 6-well tissue culture plates in the presence of MEMα medium supplemented with 10% FBS. On days 3-5 after differentiation, the differentiating mGSCs were harvested by 0.05% trypsin and stained with PE-conjugated anti-*PdgfR-α* and anti-*Flk1* antibody. The stained cells were assessed by FACS analysis. All data are represented as a percentage (%) of the total population.

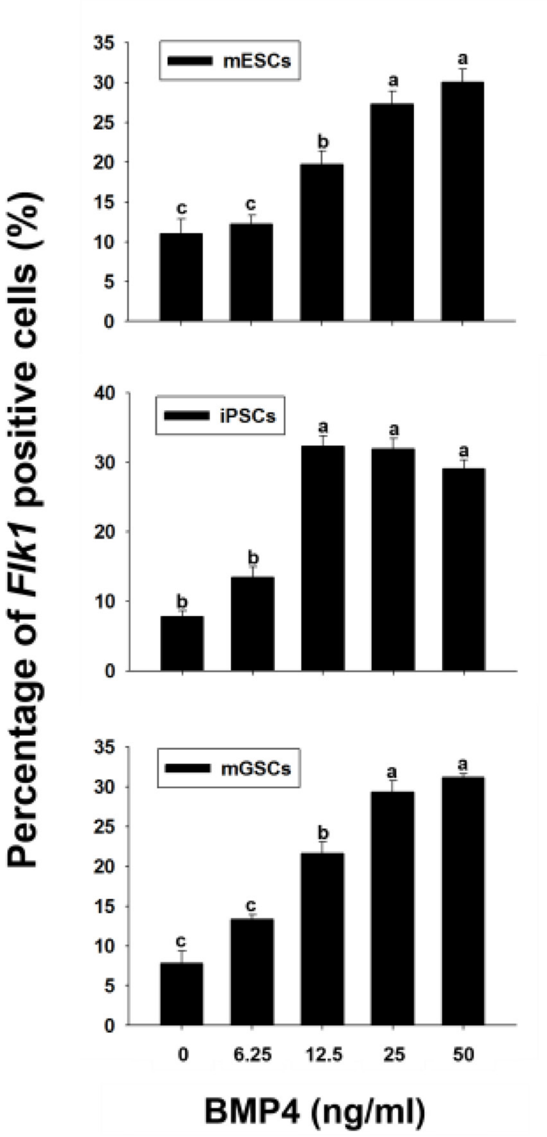

Supplementary Figure 3: Effect of BMP4 on *Flk1* expression. Cells were cultured in MEMα supplemented with 10% FBS. Flow cytometric analysis of anti-FLK1 antibody selected cells. The vertical bar graph represents protein expression of $FLK1^+$ selected cells treated with APC-conjugated antibody was compared with that of isotype control (mean ± SEM; n = 3). Means with different letters are significantly different (P < 0.05).

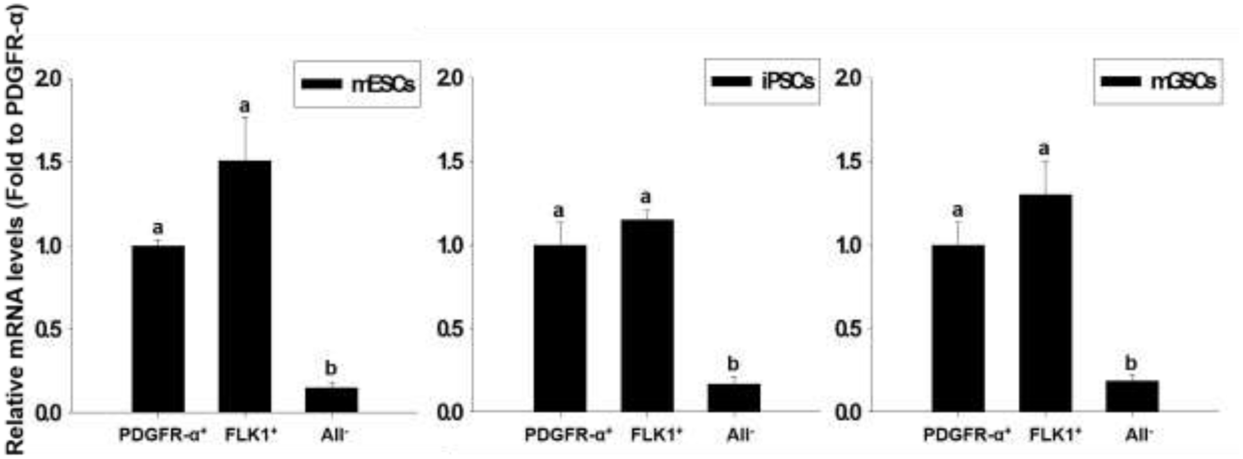

Supplementary Figure 4: Relative expressions of mature cardiac-specific gene, *cTnT*, in PDGFR-α$^+$, FLK1$^+$ and all$^-$ cell populations. Following FACS mediated sorting of PDGFR-*α*$^+$ and FLK1$^+$cell populations, the cells were exposed to N2/B27 medium containing 30 ng/mL bFGF and 10 ng/mL VEGF. The levels of *cTnT* gene expression was evaluated by normalizing to the value for PDGFR-*α*$^+$ expression (mean ± SEM; n = 3). Means with different letters are significantly different (P < 0.05).
